# A Familiar(ity) Problem: Assessing the Impact of Prerequisites and Content Familiarity on Student Learning

**DOI:** 10.1371/journal.pone.0148051

**Published:** 2016-01-29

**Authors:** Justin F. Shaffer, Jennifer V. Dang, Amanda K. Lee, Samantha J. Dacanay, Usman Alam, Hollie Y. Wong, George J. Richards, Pavan Kadandale, Brian K. Sato

**Affiliations:** 1 Department of Developmental and Cell Biology, University of California Irvine, Irvine, California, United States of America; 2 Department of Molecular Biology and Biochemistry, University of California Irvine, Irvine, California, United States of America; University of Guelph, CANADA

## Abstract

Prerequisites are embedded in most STEM curricula. However, the assumption that the content presented in these courses will improve learning in later courses has not been verified. Because a direct comparison of performance between students with and without required prerequisites is logistically difficult to arrange in a randomized fashion, we developed a novel familiarity scale, and used this to determine whether concepts introduced in a prerequisite course improved student learning in a later course (in two biology disciplines). Exam questions in the latter courses were classified into three categories, based on the degree to which the tested concept had been taught in the prerequisite course. If content familiarity mattered, it would be expected that exam scores on topics covered in the prerequisite would be higher than scores on novel topics. We found this to be partially true for “Very Familiar” questions (concepts covered in depth in the prerequisite). However, scores for concepts only briefly discussed in the prerequisite (“Familiar”) were indistinguishable from performance on topics that were “Not Familiar” (concepts only taught in the later course). These results imply that merely “covering” topics in a prerequisite course does not result in improved future performance, and that some topics may be able to removed from a course thereby freeing up class time. Our results may therefore support the implementation of student-centered teaching methods such as active learning, as the time-intensive nature of active learning has been cited as a barrier to its adoption. In addition, we propose that our familiarity system could be broadly utilized to aid in the assessment of the effectiveness of prerequisites.

## Introduction

Typical undergraduate STEM curricula are sequential, requiring the completion of certain prerequisites prior to enrolling in subsequent courses. A theoretical reason for this arises from the constructivist perspective of learning, which suggests that individuals utilize past experience in making sense of new ideas or concepts [1–5]. Material covered in prerequisite courses may provide students with targeted exposure to new information that they may use to aid their understanding of novel phenomenon introduced in a later course. In this way, a layered curricular structure, in theory, facilitates development of students into more knowledgeable, scientifically literate individuals. In addition, there may be non-pedagogical reasons to include prerequisites in a curriculum. For example, required prerequisites allow administrators to have a more accurate estimate of future course enrollments (i.e. If 100 students take BIO1, then we know 100 or fewer will be enrolling in BIO2 the next academic term) and the necessary resources to allocate. While the latter purpose is readily demonstrable, there has been little done to rigorously assess the idea that completion of prerequisites is important for success in later courses.

A few prior studies have examined the impact of prerequisites on the individual course level. McRae identified a statistically significant correlation between the grade earned in a prerequisite undergraduate organic chemistry course and the grade earned in a chiropractic biochemistry course at the same institution [6]. Similarly, Forester et al. saw that students who had taken any undergraduate histology or anatomy course earned a significantly higher course grade in a medical school histology or anatomy course [7]. On the other hand, Wright et al. found that students who completed an organic chemistry prerequisite course earned similar grades in a biochemistry class as their peers who did not take the prerequisite [8]. And Steele and Barnhill illustrated that students in a medical school genetics course earned similar exam scores regardless of whether they had previously enrolled in an undergraduate genetics course [9]. Caplan et al. conducted a broader examination of medical school prerequisites including anatomy, biochemistry, histology and embryology, and their relationship with the corresponding medical school course, and only found a correlation between grades earned in undergraduate and graduate biochemistry [10]. In addition, work has been done to examine the impact of prerequisites on program level success (i.e. major GPA, graduation rates), both at the undergraduate and graduate levels. Similar to the above studies, these assessments have produced mixed results regarding the connection between prerequisites and program success [11–15].

These examples highlight not only the wide range of findings concerning the value of prerequisite courses, but the differing ways in which this question is addressed. While the most straightforward means is to examine the performance of students who have or have not completed a prerequisite course, this limits assessment only to recommended prerequisites, as opposed to required prerequisites, which are the basis of many STEM programs. Another method to assess the value of prerequisites is to identify whether there is a correlation in grades earned between a prerequisite and associated course. This would assume though that significant fractions of the prerequisite and latter courses overlap, in particular the exam questions, as these usually make up a large portion of course grades. It also does not take into consideration that students who earn high GPAs must be performing well in the majority of their courses—even in unrelated ones—thus limiting the conclusions that can be drawn regarding prerequisites.

To address the hypothesis that content covered in prerequisites is important for success in future courses, we focused on the specific content presented in pre and post-requisite courses with an innovative system centered on content familiarity. As prerequisites are meant to prime future learning, we would expect that students would be more knowledgeable and earn higher grades on exam questions covering material that was taught in a prior course.

For our study, we investigated student exam performance in a Molecular Biology (MB) course in the context of a closely related prerequisite Genetics (G) course as well as a Human Anatomy (A) course and its Human Physiology (P) prerequisite. Based on the hypothesis that content coverage in prerequisite courses improves student learning in future courses, we would expect that in the later course (MB/A), students would perform better on exam questions covering content they were familiar with from the prerequisite course (G/P), compared to questions that addressed novel content matter. Our results demonstrate that this hypothesis is only partially correct, highlighting the need for more rigorous prerequisite and curriculum assessment. By using our novel familiarity system, it is possible to generate data that can be utilized to evaluate a program and potentially drive undergraduate curriculum transformation.

## Materials and Methods

### Description of Data and Analysis

This study was conducted at a large, public, R1 research university in the western United States and focused on a Genetics course (G) that is a prerequisite for a Molecular Biology course (MB) and a Human Physiology course (P), which is a prerequisite for a Human Anatomy course (A). Both G and MB are lower division courses required for Biological Sciences and Pharmaceutical Sciences majors, and are generally taken by second year students. Both P and A are upper division courses, which act as electives for Biological Sciences and Pharmaceutical Sciences majors and are generally taken by third and fourth year students. Additionally, the A course is required for second year Nursing Science students. Descriptive data regarding the students in these courses are presented in Table 1.

**Table 1:** Descriptive statistics of the students in the study courses.

	Mol Bio Spring 2014 (ten weeks)	Mol Bio Spring 2015 (ten weeks)	Mol Bio Total n (%)	Anatomy Spring 2014 (ten weeks)	Anatomy Summer 2014 (five weeks)	Anatomy Spring 2015 (ten weeks)	Anatomy Total n (%)
**Number of Students (n)**	245	126	371	77	14	111	202
***Gender***							
Male	88	72	160 (43.1)	22	6	40	68 (33.7)
Female	156	52	208 (56.1)	53	8	71	132 (65.3)
Unknown	1	2	3 (1.0)	2	0	0	2 (1.0)
***Ethnicity***							
White	34	33	67 (18.1)	13	2	20	35 (17.3)
Asian	162	65	227 (61.2)	58	8	70	136 (67.3)
Hispanic/Latino	39	24	63 (17.0)	3	4	17	24 (11.9)
African American	6	1	7 (1.9)	1	0	4	5 (2.5)
Unknown	4	3	7 (1.9)	2	0	0	2 (1.0)
**Average College GPA (±SD)**	3.27 ± 0.36	3.21 ± 0.43	3.24 ± 0.39	3.37 ± 0.33	3.22 ± 0.36	3.39 ± 0.35	3.37 ± 0.34
**Average Percentage on Study Exam Questions (±SD)**	56.9 ± 8.3	59.2 ± 10.5	57.7 ± 9.1	80.6 ± 19.4	79.7 ± 20.0	74.7 ± 17.8	78.6 ± 19.2

Demographic data of students enrolled in sections of MB and A. GPA is reported on a 4.0 scale. Exam score performance is reported as a percentage with 100% being the maximum possible.

For this analysis, student exam data were collected from MB during the Spring 2014 and Spring 2015 academic quarters taught by the same instructor (BKS) and A during the Spring 2014, Summer 2014, and Spring 2015 academic quarters taught by the same instructor (JFS). Data were only analyzed for students who took both the pre and post course at our institution (i.e. students who took both G and MB or P and A at our institution). During the study quarters, both MB and A were taught in a high structure format [16, 17] with multiple exams (3 midterm exams and a comprehensive final exam), assigned textbook readings and associated pre-lecture reading assignments, active lecture periods, weekly online homework assignments (MB) or quizzes (A), and active discussion sections (MB) or laboratory sections (A). For each course in each quarter, questions from the midterm (MB) or final (A) exams were characterized based on G or P course familiarity. For MB, this included a total of 36 exam questions from 2014 and 2015 each. For A, this included a total of 38 questions from Spring 2014, 38 questions from Summer 2014, and 18 questions from Spring 2015. Question familiarity was characterized as described in the following section.

This study was performed with approval from the University of California, Irvine Institutional Review Board (HS# 2012–9191, HS#2013–9959). As this was treated as exempt research (and as approved by the IRB), all students in the previously noted courses were automatically enrolled in the study, and no written consent was obtained. Those who did not want to participate emailed an independent party to have their data removed from analysis. No students chose to opt out of the study.

### Familiarity Designation

A familiarity designation was assigned to each exam question in the MB and A courses in three distinct ways, including (1) analysis of the prerequisite course lecture slides, (2) analysis by the prerequisite course instructor, and (3) by a focus group of students who had just completed the prerequisite course (MB only). In each manner of assigning familiarity, a common definition for each level of familiarity was used. Exam questions deemed to be very familiar (VF) were those that students should have been capable of answering based on the material presented in the G or P prerequisite. Questions based on content that was covered in G or P, but not to a sufficient degree that would allow the students to answer the MB or A exam question, were classified as familiar (F). And questions on content not discussed in G or P were categorized as not familiar (NF).

1. **Lecture Slide designation**: Lecture slides were obtained from G and P course instructors and members of the research team independently categorized each question based on the content of the lecture slides. Different sets of study team members analyzed the MB (authors JVD, AKL, SJD, UA, BKS) and A (JFS, HYW, GJR) lecture slides. There was initial agreement among each set of reviewers for over 75% of the questions. For questions where familiarity was not categorized identically, the study team members debated until a consensus decision was reached.
2. **Instructor designation**: G and P instructors (who were blind to the purpose of the study) were presented with a fraction (twelve questions) of the MB or A exam questions and the familiarity definitions, and were asked to characterize each of the questions based on their perspective. Instructors were not presented with all of the questions due to the time intensive nature of categorizing all the questions.
3. **Student Focus Group designation**: For the G/MB analysis, a focus group of nine undergraduates who completed the G prerequisite course roughly one month prior, volunteered to view 2014 MB exam questions and to rate their familiarity based on their recent G course. The students were not informed of the study hypothesis nor were they aware that the questions were from the MB course. The focus group was presented with 34 questions and the familiarity definitions. Each student individually reported their perceived familiarity based on these definitions using the iClicker student response system. For analysis purposes, familiarity for a question was assigned if at least 6 of the 9 students agreed on the familiarity ranking. In cases where this 2/3 agreement was not met, the question was removed from the analysis. Based on these guidelines, students categorized 26 of the 34 possible questions.

### Data Analysis

MB and A exam questions were segregated based on the assigned familiarity rating, and the average percent correct on questions in each familiarity category was determined. The Shapiro-Wilk test was used to test normality of the data from each course. The MB data were normally distributed so differences in performance between questions of varying familiarity were determined by first performing a one-way ANOVA examining mean score on questions in each familiarity group. Tests identifying a p value lower than 0.05 were then analyzed using Tukey’s test, which allowed for simultaneous comparisons between each group of questions (VF vs. F, F vs. NF, VF vs. NF). The A data were not normally distributed so differences between familiarity categories were determined by the Kruskal-Wallis test with pairwise comparisons made using the Wilcoxon signed-rank test and the Bonferroni correction. To take into consideration differences in cognitive challenge among questions, we controlled for the Bloom’s level [18, 19] of each question (individual questions ranged from Bloom’s level 1 (knowledge) to 6 (synthesis)), by building a multiple regression model with question performance as the response variable. In the model, Bloom’s level and Familiarity were used as independent variables. Each Bloom’s level (1–6) was treated as a categorical value, as was familiarity (VF, F, NF). The study team who has experience “Blooming” exam questions determined a question’s Bloom’s level with universal agreement on over 80% of questions [18]. Familiarity was designated as described above. Two regression models were run for each analysis with Bloom’s level 1 as the baseline along with either F or NF familiarity. Tables with summaries of the regression model coefficients are presented in Tables A-H in S1 File. A descriptive table containing average student performance and average Bloom’s level for questions of each familiarity category can be found in Table 2.

**Table 2:** Descriptive statistics of the questions analyzed for study purposes.

Course/Familiarity Designation Method		VF	F	NF
MB (2014)/Lecture Slides	n (number of questions)	9	18	9
	Average Score (%) (±SD)	75.3 ± 16.9	50.6 ± 22.8	56.1 ± 18.4
	Average Bloom’s	2.4	3.4	2.9
MB (2014)/Instructor	n	2	4	6
	Average Score (%) (±SD)	74.7 ± 19.7	64.8 ± 16.1	54.9 ± 22.2
	Average Bloom’s	2.0	2.3	3.3
MB (2014)/Focus Group	n	11	8	7
	Average Score (%) (±SD)	63.7 ± 28.9	50.0 ± 15.1	62.6 ± 22.1
	Average Bloom’s	2.6	3.4	3.3
MB (2015)/Lecture Slides	n	12	11	13
	Average Score (%) (±SD)	62.2 ± 21.3	55.4 ± 18.4	60.4 ± 21.5
	Average Bloom’s	2.8	3.5	3.3
A (2014 Spring) /Lecture Slides	n	5	12	21
	Average Score (%) (±SD)	91.5 ± 8.6	83.5 ± 14.6	77.9 ± 22.5
	Average Bloom’s	1.1	1.2	1.1
A (2014 Summer)/Lecture Slides	n	10	9	19
	Average Score (%) (±SD)	94.7 ± 9.5	82.0 ± 24.1	72.1 ± 25.4
	Average Bloom’s	1.3	1.4	1.1
A (2015 Spring)/Lecture Slides	n	3	2	13
	Average Score (%) (±SD)	92.4 ± 7.4	73.1 ± 13.8	66.9 ± 21.8
	Average Bloom’s	1.3	1.8	1.2
A (2014 Spring & Summer)/ Instructor	n	2	4	6
	Average Score (%) (±SD)	98.4 ± 2.7	84.0 ± 18.1	86.5 ± 14.2
	Average Bloom’s	1.0	1.6	1.1

Data regarding the exam questions from each of the study courses. The descriptive statistics include the number of questions in each familiarity category (VF, F, NF) by the indicated familiarity characterization method (lecture slides, instructor, focus group), the average student score on questions in each familiarity category, and the average Bloom’s level. Average score is reported as a percentage with 100% being the maximum possible. Average Bloom’s is reported on a 1–6 scale with Blooming conducted by the study team (as reported in the methods)

## Results

### The Impact of Concept Familiarity on Learning

**Fig 1:**
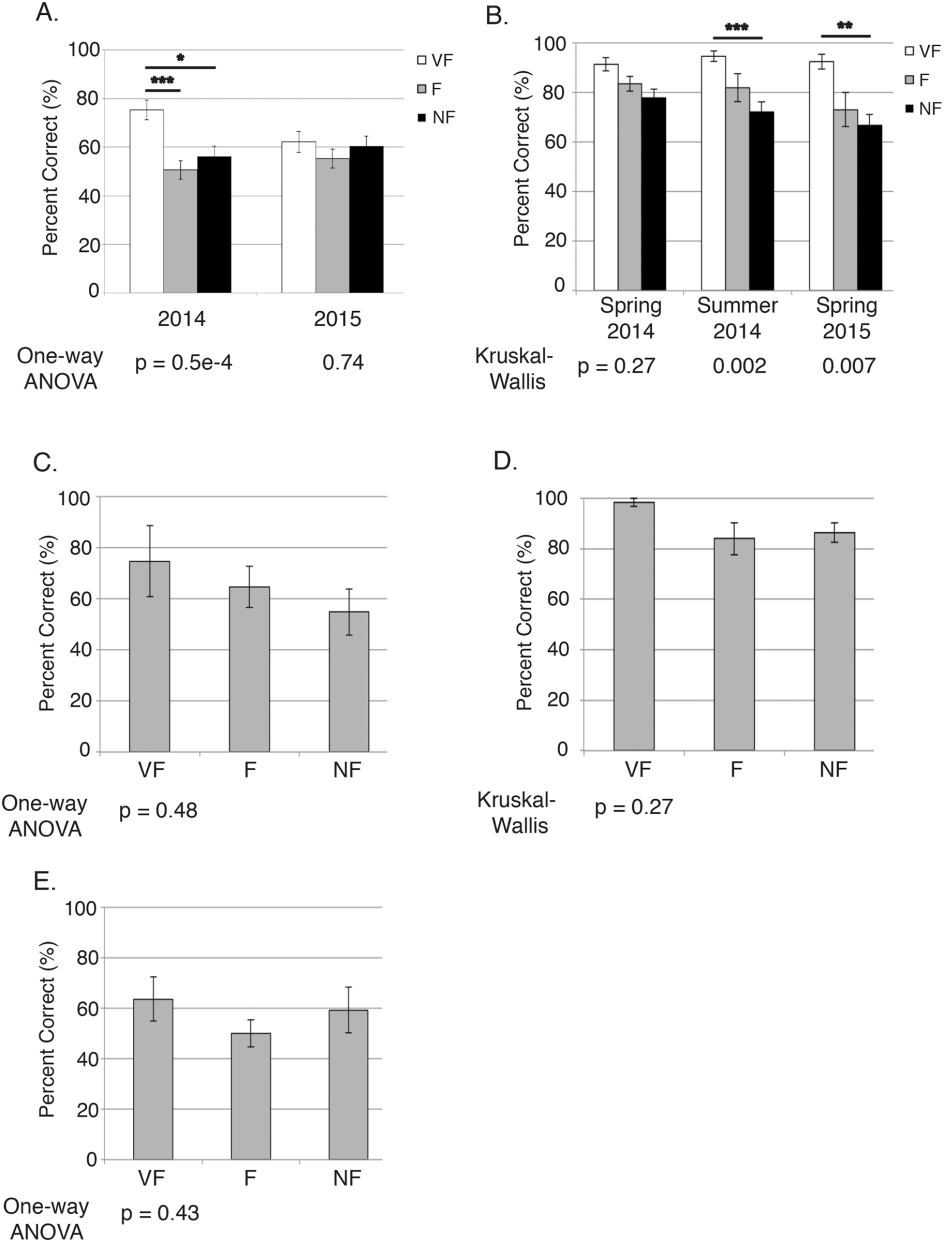
Student performance on exam questions in the context of question familiarity. Student performance on **(A)** Molecular Biology (MB) and **(B)** Human Anatomy (A) exam questions sorted by familiarity according to prerequisite course lecture slides. For the MB analysis, exam questions were analyzed from both the 2014 and 2015 courses and significant differences were determined using a one-way ANOVA followed by Tukey’s test (comparing VF vs. F, F vs. NF, and VF vs. NF performance). Because the A data were not normally distributed, they were analyzed with the Kruskal-Wallis test and the Wilcoxon signed-rank test and Bonferroni correction. * *p* ≤ 0.05, ** *p* ≤ 0.01, *** *p* ≤ 0.001. Student performance on twelve randomly selected 2014 **(C)** MB and **(D)** A exam questions sorted by familiarity according to instructors of the prerequisite courses. **(E)** Student performance on 2014 MB exam questions sorted by familiarity according to a focus group consisting of nine former G students. Question familiarity was characterized in each case as described in the methods. Average performance between categories was not significantly different for C-E as determined using one way ANOVA **(C, E)** or Kruskal-Wallis **(D)** tests. For A-E, mean performance values for questions in each category are indicated along with the standard error of the mean (SEM).

We first characterized the familiarity of MB and A exam questions using lecture slides from the prerequisite courses. When segregating exam questions by this characterization and assessing performance on questions of each familiarity category, students scored higher on VF questions in the majority of sections analyzed relative to F or NF questions, yet universally there was no significant difference observed between questions on F and NF material (Fig 1A and 1B). Next, the prerequisite course instructors examined a fraction of the MB/A exam questions using our familiarity definitions. Based on this segregation of questions, there was no significant difference in performance of questions in different familiarity groups in contrast to the VF/F or VF/NF distinction seen with the lecture slide analysis, although the trend of higher performance on VF questions remained (Fig 1C and 1D). And finally, as instructor and student perspectives of what is taught in a given course may vary, we also sought the opinion of students who had recently completed the G prerequisite. Similar to the analyses performed with the instructor familiarity designation, there was no significant difference in performance on questions across the different familiarity levels (Fig 1E). Overall, the trend of increased performance on VF over F or NF questions was observed for all analyses (although statistically significant only for a fraction of the designations), while all methods demonstrated that performance on F questions was not significantly different from performance on NF questions.

One consideration in this analysis is that the questions in each familiarity category are fundamentally different, both in terms of difficulty and the specific type of cognitive process required to answer them. As of yet, there is not a well-established difficulty scale that can be assigned independent of student performance, which as our response variable cannot be part of this designation. But we can utilize Bloom’s taxonomy [18, 19] to see whether it is an underlying factor in question performance. Multiple regression analysis was performed to examine question performance with both Bloom’s level and familiarity as factors (Tables A-H in S1 File). Bloom’s level is a strong factor in student performance, with scores becoming progressively lower with increasing Bloom’s level, especially for higher order questions. While the trend of increased VF performance relative to F or NF performance was still observed (Tables A-H in S1 File), performance on F and NF questions did not significantly differ when controlling for Bloom’s level (Table 3). This illustrates that students may be better able to answer questions on VF content independent of cognitive challenge, while there is no advantage for F concepts compared to NF concepts even when controlling for differences in Bloom’s level (Tables A-H in S1 File).

**Table 3:** Summary comparison of performance on F and NF questions only.

Course (Year)	Familiarity Designation	Estimate (+/- SEM)	P value
MB (2014)	Lecture Slides	0.00 (0.06)	0.97
MB (2014)	Instructor	0.03 (0.15)	0.86
MB (2014)	Focus Group	0.08 (0.11)	0.44
MB (2015)	Lecture Slides	0.05 (0.06)	0.45
A (Spring 2014)	Lecture Slides	-0.05 (0.05)	0.36
A (Spring 2014)	Instructor	0.04 (0.09)	0.19
A (Summer 2014)	Lecture Slides	-0.12 (0.07)	0.07
A (Spring 2015)	Lecture Slides	-0.05 (0.11)	0.67

A subset of the summary data from eight independent multiple regression models of Molecular Biology (MB) and Human Anatomy (A) exam question performance analyzed in the context of Bloom’s level (BL1-6) and familiarity (VF/F/NF). The impact of F versus NF question designation on exam question performance for each of the eight models is indicated on the table. For each of the models, the baseline values are Bloom’s level 1 and F familiarity. The estimate highlights the increase or decrease in scores (out of 100% presented in decimal form) for NF questions relative to F. The estimate, standard error of the mean, and p values are indicated for each comparison of F and NF questions. Complete data (including VF, F and NF question performance and differences across Bloom’s level) from all of the regression models are presented in Tables A-H in S1 File.

### Comparison of Exam Question Familiarity Rankings by Method

**Fig 2:**
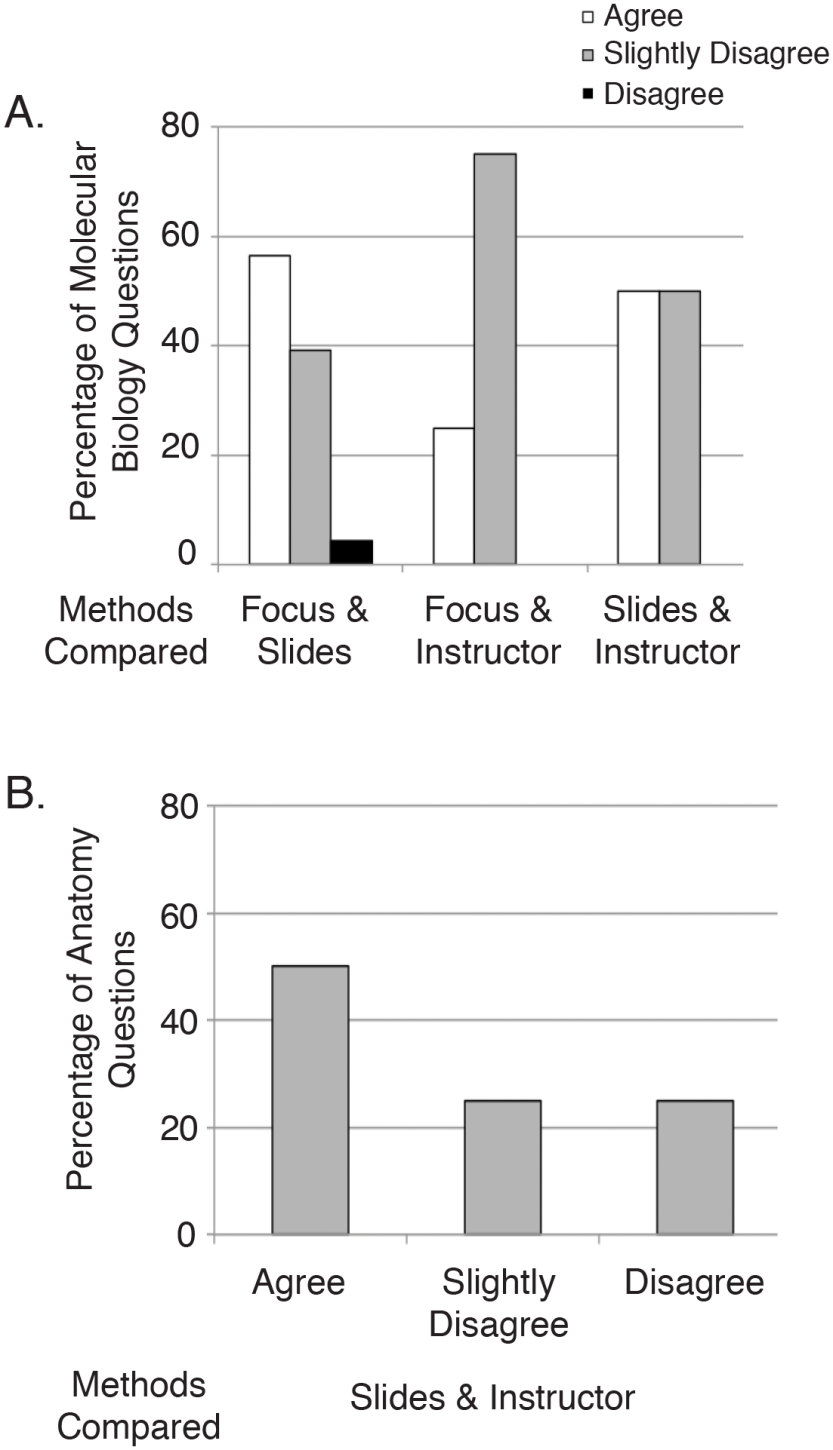
Level of agreement between different familiarity designation methods. **(A)** Question familiarity assignments were compared between the three methods, G lecture slides, a G instructor, and a student focus group for Molecular Biology (2014 exam data) and **(B)** between P lecture slides and P instructors for Human Anatomy (2014 Spring and Summer exam data). Familiarity assignments for each question as designated by the indicated two methods were compared to each other, and the fraction of questions in each group are noted. Agree, slightly disagree, and disagree were defined as described in the methods.

One of the reasons that three distinct methods were utilized to determine question familiarity was that stating what a student should know is a difficult proposition. We compared each method to the other two, noting whether the question was characterized identically (agree), whether the familiarity designation was off by one level (for example, VF by lecture slides but F by instructor, noted as slightly disagree), or whether one method rated a question as VF while another selected NF (disagree). While the number of ratings that agreed outweighed those that disagreed, it is clear that none of the methods fully agreed with another for both disciplines examined (Fig 2A and 2B). Despite these differences, using each method results in the same conclusion that students did no better on F questions than NF questions (Table 3 and A-H in S1 File).

## Discussion

The premise behind a prerequisite is that it provides an opportunity to present students with a given concept or skill, which can then be used as the starting point to build upon in a later course. In other words, future student learning should be enhanced if a topic is covered in a prerequisite as opposed to if it was not. Given the prevalence of prerequisites in STEM curricula, it is surprising that there is so little evidence supporting the effectiveness of these courses in promoting student learning in later courses. The design and implementation of such courses has therefore mostly relied on implicit assumptions that have not been tested, and which are not backed by evidence. The most obvious comparison of examining performance of students who have or have not completed a prerequisite limits the scenarios in which assessment can occur. Merely examining overall student grades in two related courses is also problematic, since this does not distinguish between effects of the prerequisite from the effects of overall student achievement. Thus, we propose that assessing specific concepts and their familiarity from one course to the next can be employed as a starting point for assessment of any prerequisite.

We tested the hypothesis that being exposed to content in the prerequisite courses G and P would allow students to better answer MB and A questions on related material. Surprisingly, students only earned higher scores on VF questions relative to F or NF questions (and this was only statistically significant in certain study scenarios, Fig 1 and Tables A-H in S1 File), but performance on F questions was never significantly higher than NF questions (Fig 1 and Table 3). We believe this distinction is important, as a common challenge for instructors is how to fit large amounts of content into a given course: the breadth versus depth argument [20–22]. A popular belief is that all the material is important, and even topics that do not receive as much time as they may deserve need to be presented for the benefit of students’ future learning [23]. Our data imply that benefits may only be seen for material covered extensively, and that rather than concern ourselves with the quantity of topics in a course, we should instead pare this down and focus on the depth of coverage. This is supported by an analysis examining the breadth versus depth argument in the context of high school science courses and success in related college courses [24]. In this study, students who reported spending at least one month on a major topic listed in the survey earned higher grades in their college science courses. On the other hand, those who reported covering all of the major topics listed on the survey did not see a similar effect on their college science grades. Our data can also be used as a piece of evidence against a common concern for instructors switching from a traditional to active teaching style, that incorporation of new classroom activities uses up time normally dedicated to delivering course material [25, 26]. Our data imply that superficially discussed content can be removed as it may not result in improved future learning. The available course time could then be replaced with activities that allow students to critically think about the topics of greater importance. The fact that similar results were observed with two distinct biology disciplines reinforces this idea.

One potential concern with our analysis is how familiarity was defined and categorized. It is our opinion that a binary system consisting of only familiar or not familiar is not sufficiently diverse, as there are wide ranges, both in terms of time and emphasis placed, in which a topic can be discussed in class. While more than three familiarity categories is an option, increasing the number of levels would likely require each to be defined in a more quantitative manner (such as time discussed in lecture or textbook pages assigned), rather than the more qualitative approach that we took.

A related question is what is the “best” way to define familiarity, or how do we gauge what a student should have learned in a course? This could potentially be viewed from the instructor or student standpoint, although past research has shown that these groups can possess differing perspectives of a classroom environment or the associated learning that is occurring [27–30]. This may in part be due the fact that novices (students) and experts (instructors) process material differently [31–33]. Another possibility is for an independent third party to use instructor prepared material, such as textbook reading assignments or lecture slides, as we did in this study. While free of the above biases, this presents another potential issue, as those reviewing the course materials can be forced to extrapolate what is present in the slides to fit an exam question. In order to increase the rigor in categorizing questions by familiarity, we decided to use multiple methods. We believe this is valid, as our conclusions were similar regardless of the familiarity ranking method used.

At best, our data imply that students perform better on exam questions covering VF content, yet certain familiarity ranking methods resulted in no significant difference in performance for these questions in both the molecular biology and anatomy courses. This alludes to an alternative conclusion, that there is little value to prerequisites in regards to content familiarity, thus raising broader questions regarding learning and assessment. The relationship between prerequisites and related courses must involve multiple levels of alignment between instructors, including learning objectives, course content, and assessment. While our study focused on content familiarity, the manner and context in which this content is presented or applied also should be taken into consideration when evaluating prerequisites. One can view a prerequisite as a course that offers students resources for thinking about a topic, but that the appropriate activation of such resources at a future time is necessary for academic success [34, 35]. The manner in which this activation occurs or needs to occur relates to the expected learning outcomes and exams in the courses being assessed. If a topic is assessed with lower order cognitive skills in one course and higher order cognitive skills in another, an additional dimension is introduced that can impact student performance. In addition, work on epistemological and conceptual framing [36, 37] suggest that factors such as student bias and prior experience, and the context in which the question is presented, can impact understanding and thus performance on an exam question. Thus, content is only one consideration when designing linked courses. While these ideas are beyond the scope of this study, we believe that our data can facilitate discussion between faculty teaching related courses and can act as a starting point for future quantitative and qualitative work regarding what our students learn, retain, and transfer within a curriculum.

## Supporting Information

S1 File Molecular Biology (MB) and Human Anatomy (A) exam question performance analyzed in the context of Bloom’s level (BL1-6) and familiarity (VF/F/NF). **Tables A-H**. Individual tables represent distinct regression models looking at exam questions categorized by the indicated familiarity method. For each data set, two models were run, one with F as the baseline and one with NF. The resulting intercept and familiarity values are indicated on the table for each model. In both cases Bloom’s level 1 is the baseline and the impact of each Bloom’s level is the same regardless of which familiarity value was used as the baseline. The estimate highlights the increase or decrease in scores (out of 100% presented in decimal form) for NF questions relative to F. The estimate, standard error of the mean, and p values are indicated. * p≤0.05 ** p≤0.01 *** p≤0.001
(DOCX)
