# Supplementary material for: A Familiar(ity) Problem: Assessing the Impact of Prerequisites and Content Familiarity on Student Learning
Source: PLoS One. 2016 Jan 29;11(1):e0148051. doi: 10.1371/journal.pone.0148051 (PMC4733054; doi:10.1371/journal.pone.0148051)
Supplement: S1 File — Tables A-H. Individual tables represent distinct regression models looking at exam questions categorized by the indicated familiarity method. For each data set, two models were run, one with F as the baseline and one with NF. The resulting intercept and familiarity values are indicated on the table for each model. In both cases Bloom’s level 1 is the baseline and the impact of each Bloom’s level is the same regardless of which familiarity value was used as the baseline. The estimate highlights the increase or decrease in scores (out of 100% presented in decimal form) for NF questions relative to F. The estimate, standard error of the mean, and p values are indicated. * p≤0.05 ** p≤0.01 *** p≤0.001 (DOCX) [file pone.0148051.s001.docx]

**Table A. Multiple Regression Analysis Examining Factors Influencing Molecular Biology Exam Performance**

**Course: 2014**

**Familiarity Designation: Lecture Slides**

|  | **Estimate (+/- SEM)** | **P value** |
| --- | --- | --- |
| **Baseline: Familiar** | | |
| Intercept | 0.66 (0.08) | < 3e-12 *** |
| Very Familiar | 0.16 (0.06) | 0.02 * |
| Not Familiar | 0.00 (0.06) | 0.97 |
| **Baseline: Not Familiar** | | |
| Intercept | 0.66 (0.08) | < 5e-12 *** |
| Familiar | 0.00 (0.06) | 0.97 |
| Very Familiar | 0.16 (0.06) | 0.02 * |
| **Baseline: Bloom’s 1** |  |  |
| Bloom’s 2 | -0.01 (0.09) | 0.92 |
| Bloom’s 3 | -0.12 (0.08) | 0.12 |
| Bloom’s 4 | -0.18 (0.08) | 0.04 * |
| Bloom’s 5 | -0.33 (0.11) | < 4e-3 ** |

**Table B. Multiple Regression Analysis Examining Factors Influencing Molecular Biology Exam Performance**

**Course: 2014**

**Familiarity Designation: Instructor**

|  | **Estimate (+/- SEM)** | **P value** |
| --- | --- | --- |
| **Baseline: Familiar** | | |
| Intercept | 0.58 (0.16) | 0.01 ** |
| Very Familiar | 0.14 (0.17) | 0.46 |
| Not Familiar | 0.03 (0.15) | 0.86 |
| **Baseline: Not Familiar** |  |  |
| Intercept | 0.61 (0.20) | 0.02 * |
| Familiar | -0.03 (0.15) | 0.86 |
| Very Familiar | 0.11 (0.20) | 0.60 |
| **Baseline: Bloom’s 1** |  |  |
| Bloom’s 2 | 0.15 (0.21) | 0.52 |
| Bloom’s 3 | 0.05 (0.17) | 0.77 |
| Bloom’s 4 | -0.21 (0.23) | 0.40 |

**Table C. Multiple Regression Analysis Examining Factors Influencing Molecular Biology Exam Performance**

**Course: 2014**

**Familiarity Designation: Focus Group**

|  | **Estimate (+/- SEM)** | **P value** |
| --- | --- | --- |
| **Baseline: Familiar** | | |
| Intercept | 0.82 (0.16) | < 6e-5 *** |
| Very Familiar | 0.04 (0.10) | 0.73 |
| Not Familiar | 0.08 (0.11) | 0.44 |
| **Baseline: Not Familiar** | | |
| Intercept | 0.90 (0.16) | < 3e-5 *** |
| Familiar | -0.08 (0.11) | 0.44 |
| Very Familiar | -0.05 (0.11) | 0.69 |
| **Baseline: Bloom’s 1** |  |  |
| Bloom’s 2 | -0.18 (0.17) | 0.31 |
| Bloom’s 3 | -0.25 (0.14) | 0.09 |
| Bloom’s 4 | -0.36 (0.16) | 0.04 * |
| Bloom’s 5 | -0.62 (0.19) | < 5e-3 ** |

**Table D. Multiple Regression Analysis Examining Factors Influencing Molecular Biology Exam Performance**

**Course: 2015**

**Familiarity Designation: Lecture Slides**

|  | **Estimate (+/- SEM)** | **P value** |
| --- | --- | --- |
| **Baseline: Familiar** | | |
| Intercept | 0.80 (0.10) | < 2e-11 *** |
| Very Familiar | 0.03 (0.06) | 0.69 |
| Not Familiar | 0.05 (0.06) | 0.45 |
| **Baseline: Not Familiar** | | |
| Intercept | 0.85 (0.09) | < 8e-14 *** |
| Familiar | -0.05 (0.06) | 0.45 |
| Very Familiar | -0.02 (0.06) | 0.71 |
| **Baseline: Bloom’s 1** |  |  |
| Bloom’s 2 | -0.19 (0.11) | 0.09 |
| Bloom’s 3 | -0.26 (0.09) | < 5e-3 ** |
| Bloom’s 4 | -0.16 (0.11) | 0.13 |
| Bloom’s 5 | -0.27 (0.11) | 0.02* |
| Bloom’s 6 | -0.54 (0.17) | < 2e-3 ** |

**Table E. Multiple Regression Analysis Examining Factors Influencing Human Anatomy Exam Performance**

**Course: 2014 (Spring)**

**Familiarity Designation: Lecture Slides**

|  | **Estimate (+/- SEM)** | **P value** |
| --- | --- | --- |
| **Baseline: Familiar** | | |
| Intercept | 0.82 (0.04) | < 2e-16 *** |
| Very Familiar | 0.09 (0.07) | 0.23 |
| Not Familiar | -0.05 (0.05) | 0.36 |
| **Baseline: Not Familiar** |  |  |
| Intercept | 0.77 (0.03) | < 2e-16 *** |
| Familiar | 0.05 (0.05) | 0.36 |
| Very Familiar | 0.13 (0.07) | 0.05* |
| **Baseline: Bloom’s 1** |  |  |
| Bloom’s 2 | 0.09 (0.07) | 0.20 |

**Table F. Multiple Regression Analysis Examining Factors Influencing Human Anatomy Exam Performance**

**Course: 2014 (Summer)**

**Familiarity Designation: Lecture Slides**

|  | **Estimate (+/- SEM)** | **P value** |
| --- | --- | --- |
| **Baseline: Familiar** | | |
| Intercept | 0.85 (0.06) | < 2e-16 *** |
| Very Familiar | 0.11 (0.07) | 0.12 |
| Not Familiar | -0.12 (0.07) | 0.07 |
| **Baseline: Not Familiar** |  |  |
| Intercept | 0.73 (0.04) | < 2e-16 *** |
| Familiar | 0.12 (0.07) | 0.07 |
| Very Familiar | 0.24 (0.06) | 0.0003*** |
| **Baseline: Bloom’s 1** |  |  |
| Bloom’s 2 | -0.07 (0.06) | 0.24 |

**Table G. Multiple Regression Analysis Examining Factors Influencing Human Anatomy Exam Performance**

**Course: 2015 (Spring)**

**Familiarity Designation: Lecture Slides**

|  | **Estimate (+/- SEM)** | **P value** |
| --- | --- | --- |
| **Baseline: Familiar** | | |
| Intercept | 0.72 (0.12) | 7.4e-07 *** |
| Very Familiar | 0.20 (0.13) | 0.14 |
| Not Familiar | -0.05 (0.12) | 0.67 |
| **Baseline: Not Familiar** |  |  |
| Intercept | 0.66 (0.04) | < 2e-16 *** |
| Familiar | 0.05 (0.12) | 0.67 |
| Very Familiar | 0.25 (0.09) | 0.009** |
| **Baseline: Bloom’s 1** |  |  |
| Bloom’s 2 | 0.02 (0.08) | 0.80 |

**Table H. Multiple Regression Analysis Examining Factors Influencing Human Anatomy Exam Performance**

**Course: 2014 (Spring and Summer)**

**Familiarity Designation: Instructor**

|  | **Estimate (+/- SEM)** | **P value** |
| --- | --- | --- |
| **Baseline: Familiar** | | |
| Intercept | 0.82 (0.08) | 1.5e-09 *** |
| Very Familiar | 0.16 (0.12) | 0.19 |
| Not Familiar | 0.04 (0.09) | 0.66 |
| **Baseline: Not Familiar** |  |  |
| Intercept | 0.86 (0.04) | 1.0e-14*** |
| Familiar | -0.04 (0.09) | 0.66 |
| Very Familiar | 0.12 (0.10) | 0.23 |
| **Baseline: Bloom’s 1** |  |  |
| Bloom’s 2 | 0.02 (0.09) | 0.79 |
